# A frameshift mutation in *GON4L* is associated with proportionate dwarfism in Fleckvieh cattle

**DOI:** 10.1186/s12711-016-0207-z

**Published:** 2016-03-31

**Authors:** Hermann Schwarzenbacher, Christine Wurmser, Krzysztof Flisikowski, Lubica Misurova, Simone Jung, Martin C. Langenmayer, Angelika Schnieke, Gabriela Knubben-Schweizer, Ruedi Fries, Hubert Pausch

**Affiliations:** ZuchtData EDV-Dienstleistungen GmbH, 1200 Vienna, Austria; Chair of Animal Breeding, Technical University of Munich, 85354 Freising, Germany; Chair of Animal Biotechnology, Technical University of Munich, 85354 Freising, Germany; Clinic for Ruminants with Ambulatory and Herd Health Services at the Centre for Clinical Veterinary Medicine, Ludwigs-Maximilians-Universitaet Muenchen, 85764 Oberschleissheim, Germany; Institute of Veterinary Pathology at the Centre for Clinical Veterinary Medicine, Ludwigs-Maximilians-Universitaet Muenchen, 80539 Munich, Germany; Bayern Genetik GmbH, 85586 Poing, Germany; Institute for Infectious Diseases and Zoonoses, Ludwigs-Maximilians-Universitaet Muenchen, 80539 Munich, Germany

## Abstract

**Background:**

Low birth weight and postnatal growth restriction are the most evident symptoms of dwarfism. Accompanying skeletal aberrations may compromise the general condition and locomotion of affected individuals. Several paternal half-sibs with a low birth weight and a small size were born in 2013 in the Fleckvieh cattle population.

**Results:**

Affected calves were strikingly underweight at birth in spite of a normal gestation length and had craniofacial abnormalities such as elongated narrow heads and brachygnathia inferior. In spite of a normal general condition, their growth remained restricted during rearing. We genotyped 27 affected and 10,454 unaffected animals at 44,672 single nucleotide polymorphisms and performed association tests followed by homozygosity mapping, which allowed us to map the locus responsible for growth failure to a 1.85-Mb segment on bovine chromosome 3. Analysis of whole-genome re-sequencing data from one affected and 289 unaffected animals revealed a 1-bp deletion (g.15079217delC, rs723240647) in the coding region of the *GON4L* gene that segregated with the dwarfism-associated haplotype. We showed that the deletion induces intron retention and premature termination of translation, which can lead to a severely truncated protein that lacks domains that are likely essential to normal protein function. The widespread use of an undetected carrier bull for artificial insemination has resulted in a tenfold increase in the frequency of the deleterious allele in the female population.

**Conclusions:**

A frameshift mutation in *GON4L* is associated with autosomal recessive proportionate dwarfism in Fleckvieh cattle. The mutation has segregated in the population for more than 50 years without being recognized as a genetic disorder. However, the widespread use of an undetected carrier bull for artificial insemination caused a sudden accumulation of homozygous calves with dwarfism. Our findings provide the basis for genome-based mating strategies to avoid the inadvertent mating of carrier animals and thereby prevent the birth of homozygous calves with impaired growth.

**Electronic supplementary material:**

The online version of this article (doi:10.1186/s12711-016-0207-z) contains supplementary material, which is available to authorized users.

## Background

Bovine stature is a prototypical complex trait that is controlled by a few loci with large effects and numerous loci with small effects. Genome-wide association studies using dense molecular markers detected several quantitative trait loci (QTL) for growth-related traits in cattle [1–3]. The identified QTL account for a reasonable fraction of the phenotypic variation of bovine height [2, 4]. Sequence variants associated with mature height may also affect the size and weight of newborn calves [2, 3, 5].

Birth size and weight vary between breeds, parities and male and female calves [6, 7]. Birth weight in Fleckvieh cattle typically ranges from 38 to 45 kg [8]. Calves with a strikingly low birth weight and small size in spite of a normal gestation length are commonly referred to as “dwarfs”.

Dwarfism (DW) has been observed in several cattle breeds including Fleckvieh [9–11]. Low birth size and postnatal growth restriction are the most apparent characteristics of DW. Undersized animals may be normally proportionate and have an undisturbed general condition (i.e., proportionate DW [12]). However, DW may also be accompanied by disproportionately shortened limbs and skeletal deformities (i.e., disproportionate DW, chondrodysplasia [13]). Depending on the severity of the structural aberrations, disproportionate DW may be fatal [14, 15].

Both autosomal recessive and dominant modes of inheritance have been reported for bovine DW (e.g., [12, 15]). Causative mutations for DW were identified in Angus (OMIA 001485-9913 [16]), Dexter (OMIA 001271-9913 [14]), Tyrolean Grey (OMIA 000187-9913 [13]), Holstein-Friesian (OMIA 001926-9913 [15]) and Japanese Brown cattle (OMIA 000187-9913 [17]). However, to date, mutations causing DW have not yet been identified in Fleckvieh cattle.

Here, we present the phenotypic and genetic characterization of autosomal recessive DW in Fleckvieh cattle. The use of genome-wide association testing, autozygosity mapping and massive re-sequencing data enabled us to identify a frameshift mutation in the *Gon*-*4*-*Like* (*C. Elegans*) (*GON4L*) gene that is likely causal for the growth failure.

## Methods

### Animal ethics statement

Two animals were hospitalized at the animal clinic of Ludwigs-Maximilians-Universität München. Another two animals were pathologically examined at the Institute for Veterinary Disease Control (IVDC) of Austrian Agency for Health and Food Safety. One hospitalized calf was euthanized because of recurrent tympania with no prospect of improvement, and subsequently necropsied. Tissue samples were collected during necropsy. All affected animals result from inadvertent mating between carriers that occurred in Fleckvieh farms. No ethical approval was required for this study.

### Animals

Twenty-seven paternal half-sibs (16 males and 11 females) with strikingly low birth weight and postnatal growth restriction were inspected by breeding consultants at the age of 3 weeks to 18 months. Ear tissue samples were collected by breeding consultants and DNA was prepared following standard DNA extraction protocols.

### Genotyping, quality control and haplotype inference

Twenty-seven affected animals were genotyped with the Illumina BovineSNP50 v2 BeadChip that includes 54,609 SNPs. The per-individual call rate ranged from 98.96 to 99.60 % with an average call rate of 99.33 %. In addition, genotypes of 10,454 unaffected Fleckvieh animals that had been genotyped with the Illumina BovineSNP50 v1 BeadChip and the Illumina BovineHD BeadChip were available [18, 19]. The genotype data of cases and controls were combined and SNPs that were present in both datasets were retained for further analyses. Following quality control (minor allele frequency higher than 0.5 %, no deviation from the Hardy–Weinberg equilibrium (P > 0.0001), and per-SNP and per-individual call rates higher than 95 %), 10,481 animals (27 affected, 10,454 unaffected) and 44,672 SNPs remained for association testing. The *Beagle* software [20] was used to impute sporadically missing genotypes and to infer haplotypes.

### Haplotype-based association testing

A sliding window of 25 contiguous SNPs (corresponding to an average haplotype length of 1.42 ± 0.43 Mb) was shifted along the genome in steps of two SNPs. Within each sliding window, all haplotypes with a frequency higher than 0.5 % (N = 787,232) were tested for association with DW using Fisher exact tests of allelic association. Haplotypes with a P value less than 6.35 × 10^−8^ (5 % Bonferroni-corrected significance threshold) were considered as significantly associated.

### Generation of sequence data

Genomic DNA was prepared from a frozen semen sample of the assumed founder (DW_het_) and from an ear tissue sample of one affected animal (DW_hom_) following standard DNA extraction protocols. Paired-end libraries were prepared using the paired-end TruSeq DNA sample preparation kit (Illumina) and sequenced using the HiSeq 2500 instrument (Illumina). The resulting reads were aligned to the University of Maryland reference sequence of the bovine genome (UMD3.1 [21]) using the *BWA* software tool [22]. Individual files in SAM format were converted into BAM format using *SAMtools* [23]. Duplicate reads were marked with the MarkDuplicates command of *Picard Tools* [24]. To help identify the causal mutation, we used sequence data from another 288 unaffected animals from nine cattle breeds (Gelbvieh, Nordic Finncattle, Fleckvieh, Holstein-Friesian, Brown-Swiss, Original Braunvieh, Original Simmental, Red-Holstein, Ayrshire) that had been generated previously [25, 26].

### Variant calling and imputation

DW_hom_, DW_het_ and 288 control animals from nine cattle breeds were genotyped simultaneously for SNPs, short insertions and deletions using the multi-sample approach implemented in *mpileup* of *SAMtools* along with *BCFtools* [23]. *Beagle* phasing and imputation (see above) was used to improve the primary genotype calling by *SAMtools*. The detection of structural variants was performed on DW_hom_, DW_het_ and 203 sequenced control animals that had an average genome fold coverage greater than 10× using the *Pindel* software package with default settings [27].

### Identification of candidate causal variants

To identify mutations that were compatible with the recessive mode of inheritance of DW, all polymorphic sites within the DW-associated region were filtered for variants that met three conditions: (1) DW_hom_ was homozygous for the alternate allele, (2) DW_het_ was heterozygous and (3) all control animals were homozygous for the reference allele. Candidate causal variants were annotated using the *Variant Effect Predictor* tool [28, 29]. Sequence variants of 1147 animals from Run4 of the 1000 bull genomes project [15] were analyzed to obtain the genotype distribution of candidate causal variants in various bovine populations.

### Manual re-annotation of the bovine *GON4L* gene

A mutation in the coding sequence of the *GON4L* gene, i.e., rs723240647 was associated with DW. Since the annotation of the bovine genome may be flawed, we manually re-annotated the genomic structure of *GON4L* (ENSBTAG00000020356) based on the University of Maryland (UMD3.1) bovine genome sequence assembly [21] and the Dana-Farber Cancer Institute bovine gene index release 12.0 [30] using the *GenomeThreader* software tool [31]. The *GenomeThreader* output was viewed and edited using the *Apollo* sequence annotation editor [32].

### Validation of candidate causal variants

PCR primers were designed to analyze the polymorphism of rs723240647 using Sanger sequencing (see Additional file 1: Table S1). Genomic PCR products were sequenced using the BigDye^®^ Terminator v1.1 Cycle Sequencing Kit (Life Technologies) on the ABI 3130x1 Genetic Analyzer (Life Technologies). Genotypes for rs723240647 and rs715250609 were obtained for 3882 and 1851 Fleckvieh animals, respectively, using KASP™ (LGC Genomics) genotyping assays (see Additional file 1: Table S1).

### Clinical and pathological examination of four animals with DW

Two calves with DW were pathologically examined at the Institute for Veterinary Disease Control (IVDC) of Austrian Agency for Health and Food Safety at the age of 101 and 143 days. Another two calves with DW were referred to the animal clinic at the age of 57 and 93 days. Initial examination (including weighing) was performed upon admission. The younger calf suffered from recurrent tympania and was euthanized 4 days after admission because there was no prospect of improvement and it was subsequently necropsied. Tissue samples were collected during necropsy. The older calf was hospitalized for 400 days. Weight records were collected once a week.

### RT-PCR

Total RNA from lymph nodes, thymus, lung, heart, pancreas, liver, kidney and spleen of the euthanized animal was extracted from tissue samples using Trizol (Invitrogen) according to the manufacturer’s protocol with some modifications. After *DNase I* (Ambion) treatment, RNA was quantified using a NanoDrop ND-1000 (PeqLab) spectrophotometer, and RNA integrity was determined by RNA Nano6000 Labchip (Agilent Technologies). Complementary DNA (cDNA) was synthesized using the SuperScript IV transcriptase (Thermo Fisher Scientific). *GON4L* mRNA was examined by RT-PCR using primers 1F-GAGTCAAGCAGCTCAAACCC and 1R-AGCCAAGTCAGTTTCTCCATT, which hybridize to exons 20 and 21 and amplify a 348-bp product based on the mRNA reference sequence (NCBI accession number: XM_010802911) of the bovine *GON4L* gene. The shorter version of exon 21 was amplified using reverse primer 2R-CTCAGACTCACCCTCCTGACTC. RT-PCR was performed in 20 mL reaction volumes containing diluted first-strand cDNA equivalent to 50 ng input RNA. PCR products were loaded on 2 % agarose gels.

## Results

### Phenotypic manifestation of dwarfism

**Fig. 1:**
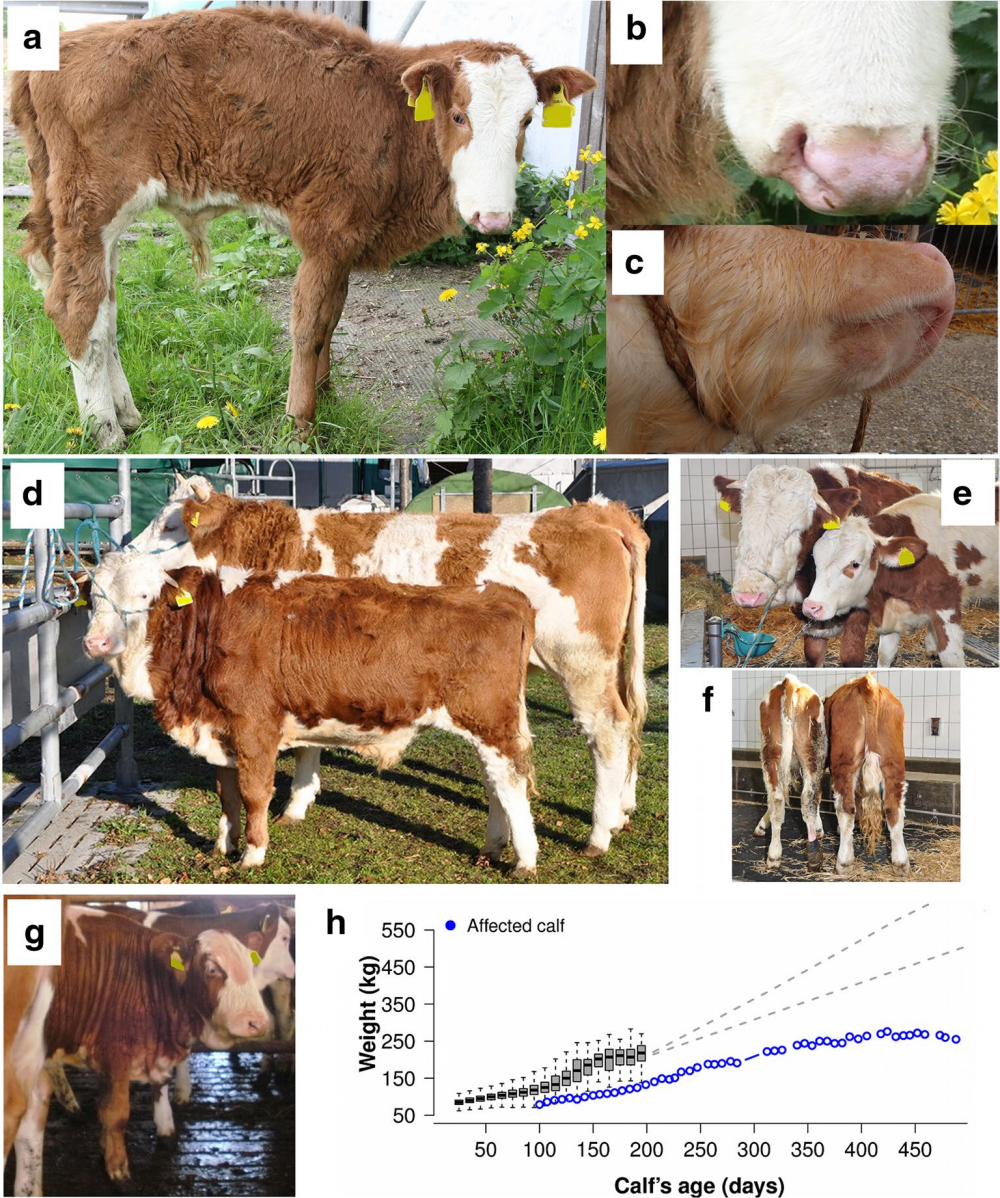
Phenotypic manifestation of dwarfism in Fleckvieh cattle. **a**–**c** A 20-week old Fleckvieh calf with a crooked back, structural abnormalities of the muzzle and brachygnathia inferior. **d** A 15-month old Fleckvieh bull with dwarfism and a healthy coeval. Note the skin flaps in the neck area. **e**, **f** The same animal as in **d** compared with a 6-month old healthy animal. Note the disproportionately large head of the affected animal (*left*) (**e**) although its height (*right animal*, height at withers: 112 cm) is similar to the nine-month younger healthy animal (*left*) (**f**). **g** An 18-month old animal with dwarfism with excessive skin in the neck area. **h** The weight of the animal shown in **d** (*blue*) is compared to the weight of 74,422 Fleckvieh calves (*grey boxes*). The *upper dotted line* represents the growth of Fleckvieh bulls observed in Geuder et al. [46]. The *lower dotted line* is a growth curve assuming an average weight gain of 1000 g/day, i.e., a lower bound estimate for the growth of Fleckvieh bulls

Twenty-seven calves (16 males and 11 females) with a strikingly low birth weight (~15 kg) and a small size in spite of a normal gestation length were detected among the descendants of an artificial insemination bull that was used for more than 290,000 inseminations. Four affected calves were clinically and pathologically examined. At the age 61, 97, 101 and 143 days, they were underweight with weight values of 42, 79, 53 and 51 kg, respectively. The calves had multiple craniofacial aberrations (i.e., brachygnatia inferior, elongated narrow heads, structural deformities of the muzzle) and spinal distortions. Wrinkled skin, areas with excessive skin and a disproportionately large head became visible during rearing (Fig. 1) and (see Additional file 2: Figure S1). Although the general condition, feed intake and locomotion of the animals were normal, their growth remained restricted. The average weight gain of an affected animal during a hospitalisation period of 400 days was only 450 g per day, i.e., less than half the weight gain of healthy Fleckvieh bulls (Fig. 1h). The growth of the sire and all dams was normal. Since both sexes were affected and most dams had a common ancestor, we hypothesized an autosomal recessive mode of inheritance. Dominant inheritance of DW was unlikely because less than 0.1 % of the progeny were affected.

### Dwarfism maps to chromosome 3

**Fig. 2:**
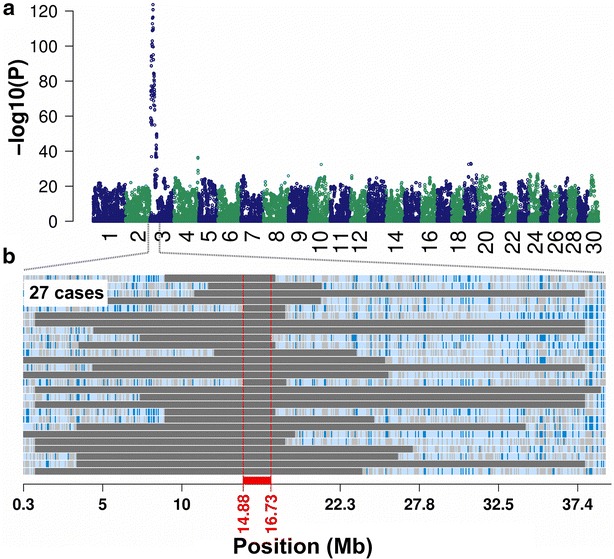
Mapping of the genomic region associated with dwarfism. **a** Association of 787,232 haplotypes with dwarfism in 27 affected and 10,454 unaffected animals. P values were obtained by calculating Fisher exact tests of allelic association. **b** Autozygosity mapping in 27 animals with dwarfism. *Blue* and *pale blue* represent homozygous genotypes (AA and BB), heterozygous genotypes (AB) are displayed in *light grey*. The *solid grey bars* represent segments of extended homozygosity in 27 animals with dwarfism. The *red bar* indicates the shared segment of homozygosity

To identify the genomic region associated with DW, 27 affected and 10,454 unaffected animals were genotyped using a medium-density genotyping array. After quality control, 44,672 SNPs were retained for genome-wide association testing. Because all affected animals were highly related with each other, the haplotype-based association study with DW revealed many significantly associated haplotypes. However, a striking association between DW and a proximal region of bovine chromosome 3 was identified (Fig. 2a). The most significant association signal (P = 2.18 × 10^−124^) resulted from two contiguous haplotypes located between 14,884,969 and 16,557,950 bp on bovine chromosome 3.

Autozygosity mapping revealed a 1.85-Mb segment (between 14.88 and 16.73 Mb) of extended homozygosity that was shared between the 27 affected animals, which corroborated a recessive mode of inheritance (Fig. 2b). The shared segment of extended homozygosity encompassed 71 transcripts/genes. However, none of them had previously been associated with DW.

Among the 10,454 control animals, 81 were heterozygous and none was homozygous for the DW-associated haplotype, which corresponded to a haplotype frequency of 0.38 %. In the recent male breeding population (birth years 2000–2012), the frequency of the DW-associated haplotype was 0.25 % (see Additional file 3: Figure S2). Haplotype frequency was considerably higher (2.6 %) in the female population because of the widespread use of an undetected carrier bull for artificial insemination [33].

Haplotype and pedigree analysis enabled us to track the DW-associated haplotype back (up to 12 generations) to an artificial insemination bull (DW_het_) born in 1959. DW_het_ was present in the maternal and paternal lineage of 21 affected animals. However, DW_het_ was not detected within the pedigree of six dams, which may be due to incomplete pedigree information and recording errors (see Additional file 4: Figure S3). The missing connection between six dams and DW_het_ may also indicate that the mutation occurred several generations before DW_het_.

### Identification of candidate causal variants for dwarfism

One affected animal (DW_hom_) and DW_het_ were sequenced to an average read depth of 13×. In addition, to help identify the underlying mutation, we exploited sequence data from 288 animals from nine breeds including 149 Fleckvieh animals. None of the 149 sequenced control animals of the Fleckvieh population carried the DW-associated haplotype.

Multi-sample variant calling within the 1.85-Mb segment of extended homozygosity revealed 11,475 single nucleotide and short insertion and deletion polymorphisms as well as 3158 larger structural variants. These 14,633 polymorphic sites were filtered for variants that were compatible with a recessive mode of inheritance, i.e., DW_hom_ homozygous for the alternate allele, DW_het_ heterozygous and 288 control animals homozygous for the reference allele (assuming that the mutation is specific to the Fleckvieh breed). This approach revealed ten candidate causal variants for DW (Table 1), among which five were intergenic, four were located in introns of the *KCNN3*, *ADAR* and *TDRD10* genes, and one variant was located in the coding region of the *GON4L* gene (see Additional file 5: Table S2).

**Table 1:** Ten sequence variants that are compatible with recessive inheritance

Chr	Position (bp)	NCBI reference ID	Type	Ref	Alt	Affected gene	Effect
3	15,079,217	rs723240647	Indel	C	–	*GON4L*	p.E1430Kfs66
3	15,713,943*	rs524337907	SNP	G	C	–	–
3	15,713,959*	rs719431247	SNP	G	A	–	–
3	15,737,755*	rs723848297	SNP	C	T	–	–
3	15,737,992*	ss1457237026	Indel	T	–	–	–
3	15,738,245*	rs720131431	Indel	C	–	–	–
3	15,815,016*	rs723370534	SNP	G	A	*KCNN3*	Intronic
3	15,924,914*	rs717718209	SNP	G	A	*KCNN3*	Intronic
3	16,046,490*	rs720952332	SNP	T	C	*ADAR*	Intronic
3	16,131,785	rs715250609	SNP	T	C	*TDRD10*	Intronic

The chromosomal position (base pairs) of compatible variants was based on the UMD3.1 assembly of the bovine genome. The asterisks indicate eight variants that are polymorphic among 1005 animals from 28 breeds other than Fleckvieh that had been sequenced for the 1000 bull genomes project

*Ref* reference allele, *Alt* alternate allele

Eight of the ten compatible variants were excluded as being causative for DW because they segregated in 1005 animals from 28 breeds other than Fleckvieh that had been sequenced for the 1000 bull genomes project [15] (Table 1) and (see Additional file 6: Table S3). In conclusion, only an intronic variant in the *TDRD10* gene (rs715250609) and a coding variant in the *GON4L* gene (rs723240647) segregated with DW. The intron variant in *TDRD10* is unlikely to be deleterious to protein function because it is more than 4000 bp away from the most proximal splice site. Thus, we considered the coding variant in *GON4L* as the most likely causal mutation for DW.

### A 1-bp deletion in *GON4L* is associated with dwarfism

**Fig. 3:**
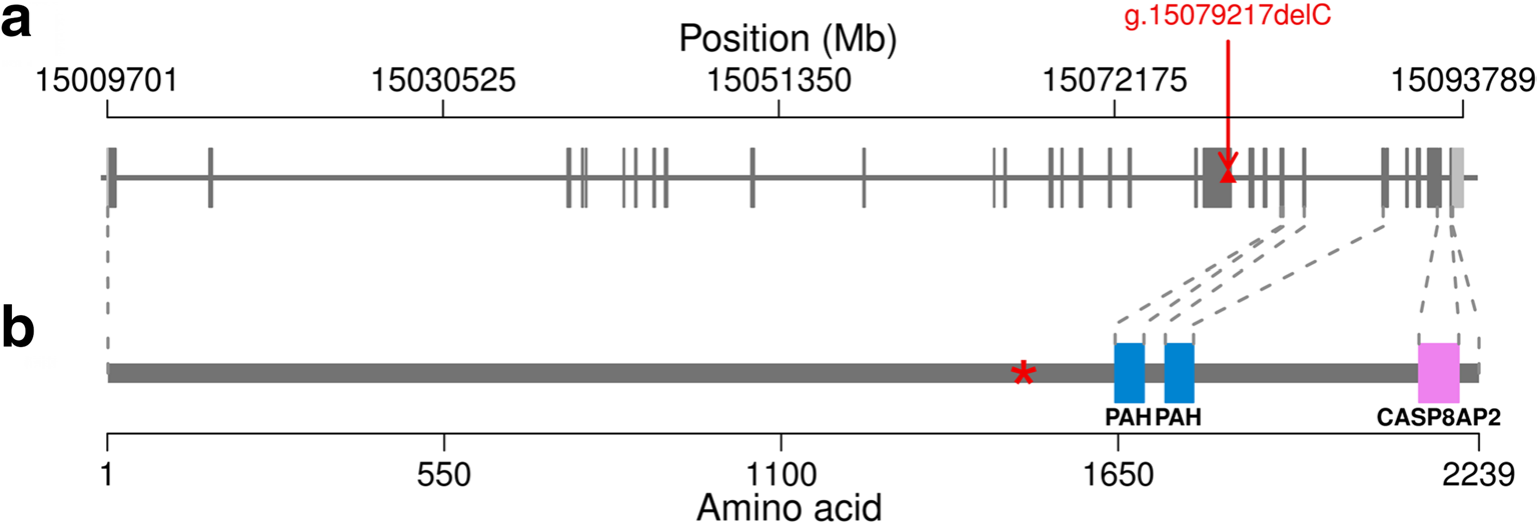
A 1-bp deletion in *GON4L* induces a premature stop codon. **a** Genomic structure of the bovine Gon-4-like encoding gene *GON4L*. *Grey* and *light grey boxes* represent exons and untranslated regions, respectively. The *red triangle* represents a 1-bp deletion (rs723240647, g.15079217delC) in exon 20 of *GON4L*. **b** The Gon-4-like protein consists of 2239 amino acids and contains highly conserved paired amphipathic helix (PAH) repeat and caspase 8-associated protein 2 myb-like (CASP8AP2) domains. The *red star* indicates the premature stop codon resulting from the 1-bp deletion

Bovine *GON4L* consists of 31 exons that encode 2239 amino acids. The variant that is compatible with recessive inheritance is a 1-bp deletion (rs723240647, g.15079217delC, ENSBTAT00000027126:c.4285_4287delCCCinsCC) in exon 20 (Fig. 3a). Sanger sequencing confirmed that DW_hom_ and DW_het_ were homozygous and heterozygous, respectively, for g.15079217delC. The deletion induces a translation frameshift that is predicted to alter the protein sequence from amino acid position 1430 onwards, and a premature translation termination codon at position 1496 (p.Glu1430LysfsX66). The Gon-4-like protein contains highly conserved paired amphipathic helix (PAH) repeats and caspase 8-associated protein 2 myb-like (CASP8AP2) domains. The mutant protein is predicted to be shortened by 745 amino acids (33 %) and to lack domains that are likely to be essential for normal protein function (Fig. 3b).

Genotypes for rs723240647 and rs715250609 were obtained for 27 affected individuals and a large number of randomly selected unaffected Fleckvieh animals using customized KASP genotyping assays (Table 2). rs723240647 was significantly associated with DW (P = 1.55 × 10^−98^). Twenty-seven calves with DW were homozygous carriers of the deletion variant, while 3855 unaffected animals were either heterozygous or homozygous for the reference allele. One animal that carried the DW-associated haplotype was homozygous for the reference allele, which may be due to a laboratory error, such as DNA sample swapping, or to haplotype recombination or imperfect genotype phasing. The intron variant in *TDRD10* (rs715250609) was almost in complete linkage disequilibrium (r^2^ = 0.98) with rs723240647 (Table 2).

**Table 2:** Genotypes for two mutations that segregate with the DW-associated haplotype

Haplotype status	Genotype at rs723240647			Genotype at rs715250609		
	C/C	C/del	del/del	T/T	T/C	C/C
Non-carrier	3581	–	–	1737	3	–
Carrier	1	82	–	–	53	–
Homozygous	–	–	27	–	–	27
Unknown	180	11	–	31	–	–

Genotypes at the rs723240647 and rs715250609 polymorphisms were obtained for 3882 and 1851 Fleckvieh animals, respectively, using custom KASP genotyping assays. The haplotype status of the animals was determined using genotypes from the Illumina BovineSNP50 BeadChip

### The deletion in *GON4L* causes intron retention and mRNA degradation

**Fig. 4:**
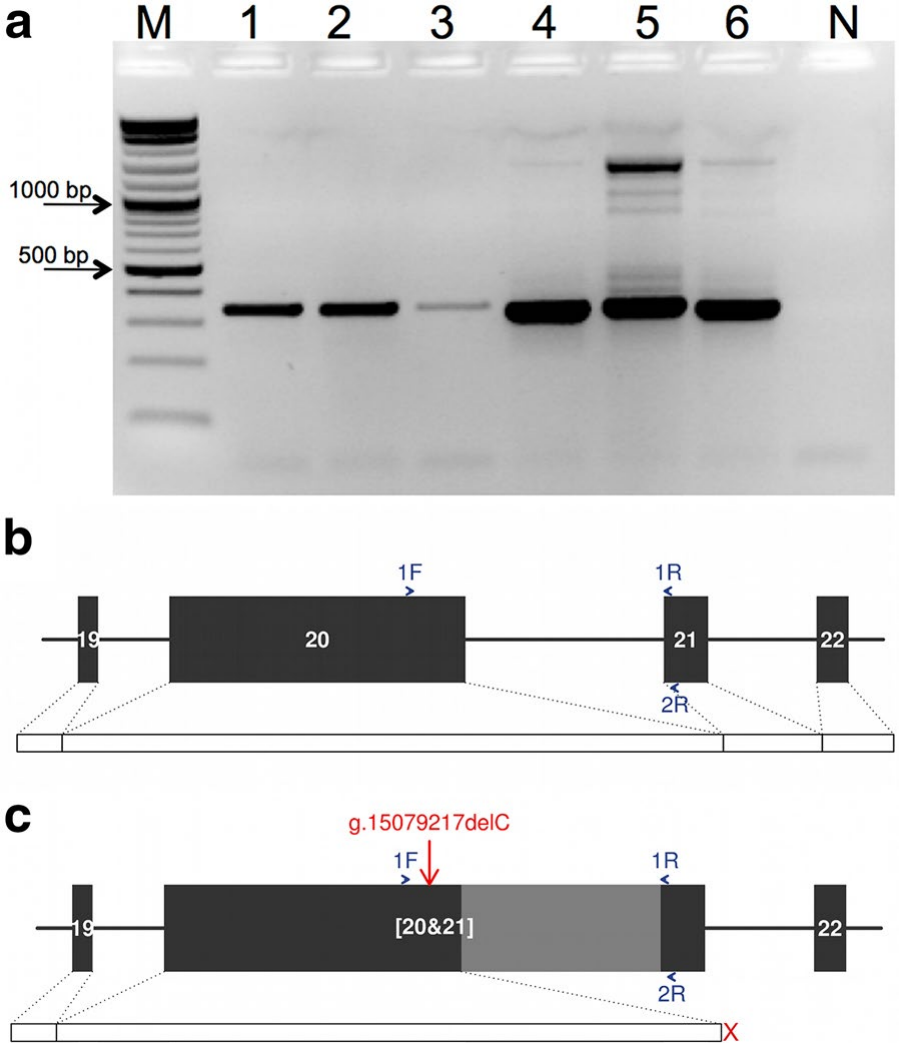
Retention of intron 20 of the *GON4L* gene in a Fleckvieh calf with dwarfism. **a** RT-PCR products were separated by 2 % agarose gel electrophoresis. *Lanes* *1*, *2*, and *3* show 348 bp RT-PCR products from lung, lymph nodes, and liver of a wild type animal. *Lanes* *4*, *5*, and *6* show 348 and 1488 bp RT-PCR products from lung, lymph nodes, and liver of a homozygous calf. *M* size marker, *N* negative RT-PCR control. **b** Schematic representation of exons 19–22 of wild type *GON4L*. **c** Schematic representation of exons 19–22 of the mutant *GON4L*. *Blue arrows* represent forward (1F) and reverse primers (1R, 2R). The *red* ‘X’ displays the premature translation termination at amino acid position 1492 resulting from the retention of intron 20. The *light grey colour* (**c**) displays intron 20

The effect of the g.15079217delC variant on *GON4L* transcription was examined by RT-PCR using RNA extracted from several tissues of a homozygous animal. Using primers located in exons 20 and 21, we obtained two RT-PCR products of 348 and ~310 bp from a wild type and a mutant homozygous animal, respectively. The longer PCR fragment corresponded to the reference mRNA sequence (NM_001192626) of the bovine *GON4L* gene. The ~310-bp PCR fragment showed a superimposed sequence of 35 bp at the 5′ end of exon 21, suggesting the presence of an alternative variant of exon 21, which is not directly associated with DW. The presence of different isoforms in the 3′ terminal end of *GON4L* in humans and cattle has been reported previously. The intensity of the signal corresponding to the alternative cDNA fragment was stronger for the mutant homozygote than for the wild type animal, which may be caused by degradation of the mutant transcript for the homozygous animal (see Additional file 7: Figure S4). We designed a reverse RT-PCR primer specific for the alternative exon 21, and obtained a unique 348-bp RT-PCR product from the wild type animal and two RT-PCR products of 313 and ~1500 bp from the mutant homozygous animal (Fig. 4). Analysis of the DNA sequence of the 348-bp wild type RT-PCR product revealed that it corresponded to the mRNA reference sequence of the bovine *GON4L* gene. Sequence analysis of the longer fragment from the mutant homozygous animal revealed that intron 20 was retained. The length of the longer PCR fragment was 1488 bp. Retention of intron 20 is predicted to introduce a frameshift mutation and to lead to a premature translation termination codon at position 1492. In conclusion, the animal homozygous for the g.15079217delC variant contains the premature translation termination codon at position 1492.

## Discussion

A 1-bp deletion in the *GON4L* gene (g.15079217delC) is associated with DW in Fleckvieh cattle. The g.15079217delC variant causes intron retention and premature translation termination and leads to a truncated protein. Compared to the wild type variant, the mutant GON4L protein is shortened by more than 30 %. RNA analysis indicated that the mutant protein variant is less abundant, which indicates that it may be degraded via nonsense-mediated mRNA decay. If the truncated protein is (partially) retained, however, its function may be compromised because it lacks domains that are possibly essential for normal protein function. Loss-of-function variants in *Udu*, a gene that is similar to *GON4L*, compromise cell cycle progression and response to DNA damage and thereby disturb embryonic growth in *D. rerio* [34–37]. In our study, the g.15079217delC variant was also associated with prenatal growth failure as evidenced by the strikingly low birth weight of homozygous calves. The phenotypic manifestation of homozygosity for g.15079217delC, i.e., pre- and postnatal growth restriction and craniofacial aberrations, resembles phenotypic patterns of human primordial DW that result from DNA repair disorders [38, 39]. Such findings suggest that disturbed growth of homozygous animals might result from defective responses to DNA damage due to impaired GON4L function. However, the actual mechanism(s) and pathway(s) that cause the extremely low birth weight and postnatal growth restriction of homozygous animals have yet to be elucidated.

Congenital disorders that manifest as growth failure have been identified in several cattle breeds. Affected calves may be born underweight or fail to thrive during rearing [26, 40–42]. The phenotypic consequences of homozygosity at g.15079217delC occur at birth. Unlike mutations in the *ACAN* and *COL2A1* genes that cause lethal disproportionate DW in cattle [14, 15], homozygosity for g.15079217delC is not fatal. Apart from large heads, affected animals were normally proportionate, and moreover, their general condition and locomotion were normal and their weight gain was constant, although considerably less than that of healthy animals. Thus, homozygosity for the g.15079217delC variant is less detrimental than, e.g., homozygosity for a mutation in *EVC2*, which compromises both growth and locomotion of affected animals [13]. Nevertheless, animals homozygous for the g.15079217delC variant are more likely to be culled at juvenile ages because of their reduced growth performance.

The g.15079217delC variant has segregated in the Fleckvieh population for more than 50 years, but due to its low frequency, DW was rarely reported. Assuming a frequency of 0.2 % for the deleterious allele, equal use of all bulls and 1,500,000 annual births in the German and Austrian Fleckvieh populations, one would expect only six homozygous calves with DW per year. However, the widespread use of undetected carriers of rare recessive alleles in artificial insemination may cause a sudden accumulation of affected calves, as our study demonstrates. Twenty-seven calves with DW were descendants from a bull that was used for more than 290,000 inseminations. The frequent use of this carrier bull resulted in a more than tenfold increase in allele frequency in the female population [33]. Our findings now enable the rapid identification of carrier animals. The g.15079217delC variant was almost in complete linkage disequilibrium with the DW-associated haplotype. Only one animal was misclassified using haplotype information, which demonstrates a high sensitivity and specificity of the haplotype-based identification of DW-mutation carriers. Since all male breeding animals are routinely genotyped with dense genotyping arrays, carriers can be readily identified using haplotype information. However, only direct gene tests will unequivocally distinguish between carrier and non-carrier animals [43]. The identification of the frameshift mutation in *GON4L* will now permit the development of customized genotyping assays to identify carrier animals. Excluding carrier bulls from artificial insemination will prevent the emergence of homozygous animals and remove from the Fleckvieh population the rare DW-associated allele within a few generations. However, sophisticated strategies are required to simultaneously consider multiple deleterious alleles in genomic breeding programs while maintaining genetic diversity and high rates of genetic gain [44, 45].

## Conclusions

A frameshift variant in the *GON4L* gene was associated with autosomal recessive proportionate DW in Fleckvieh cattle. The deleterious allele has persisted in the Fleckvieh population for more than 50 years at a very low frequency without being recognized as a genetic disorder. However, the frequent use of an undetected carrier bull for artificial insemination resulted in an accumulation of homozygous calves with DW and a tenfold increase in frequency of the deleterious allele in the female population. Our results provide the basis for the rapid identification of carrier animals and the implementation of genome-based mating strategies to avoid inadvertent carrier matings, thereby preventing the birth of homozygous calves with unsatisfactory growth performance.

## Availability of supporting data

Whole-genome sequencing data of DW_hom_ and DW_het_ were deposited in the European Nucleotide Archive (http://www.ebi.ac.uk/ena) under accession number PRJEB12832.

## Additional files

10.1186/s12711-016-0207-z Primer sequences used for the validation of two candidate causal mutations.
10.1186/s12711-016-0207-z Fleckvieh animals with dwarfism. Affected calves with a crooked back (a, b), elongated narrow heads and brachygnathia inferior (c-e). Wrinkled skin and areas with excessive skin (particularly in the neck area) became evident during rearing (c, e, g). A 19-month old animal with dwarfism and an 11-month old healthy animal (f). The head of the affected animal was disproportionately large compared to its body.
10.1186/s12711-016-0207-z Frequency of the DW-associated haplotype in 8332 Fleckvieh bulls. Grey bars and black dots represent the number of genotyped bulls and the haplotype frequency, respectively, per birth year.
10.1186/s12711-016-0207-z Analysis of pedigree records of 27 animals with dwarfism. Description: Pedigree of 21 animals with dwarfism (a). The pedigree contains only obligate mutation carriers. Red and yellow colours indicate 21 affected animals and 14 genotyped haplotype carriers, respectively. Green and blue colours indicate the sire of 21 affected animals and DW_het_, respectively. Rectangles and ovals represent male and female animals, respectively. Pedigree completeness of another six animals with dwarfism with no connection to DW_het_ on the maternal path (b).
10.1186/s12711-016-0207-z Annotation of ten candidate causal variants. The functional consequence of ten candidate causal variants was obtained from Ensembl using the *Variant Effect Predictor* tool.
10.1186/s12711-016-0207-z Genotype distribution of ten candidate causal mutations for dwarfism in 1147 animals from the 1000 bull genomes project. Alternate allele frequency and genotype distribution of ten variants in 29 breeds (homozygous animals for the reference allele | heterozygous animals | homozygous animals for the alternate allele). Blue color indicates two variants that were not polymorphic among the 1147 sequenced animals.
10.1186/s12711-016-0207-z Electrophoregram presenting the *GON4L* cDNA sequencing data with superimposed sequence at the 5’ terminal end of exon 21 (marked within a box).
