# Supplementary material for: A frameshift mutation in GON4L is associated with proportionate dwarfism in Fleckvieh cattle
Source: Genet Sel Evol. 2016 Mar 31;48:25. doi: 10.1186/s12711-016-0207-z (PMC4818447; doi:10.1186/s12711-016-0207-z)
Supplement: Supplementary file 1 — 10.1186/s12711-016-0207-z Primer sequences used for the validation of two candidate causal mutations. [file 12711_2016_207_MOESM1_ESM.pdf]

Sanger sequencing

NCBI SNP ID	Chr	Position	Genes	Forward primer sequence (5'>3')	Reverse primer sequence (5'>3')
rs723240647	3	15079217	*GON4L*	AGCTTCCCAAGTGAGGAGTC	TCCCTCTCCTCTCACCTCAA

KASP assay

NCBI SNP ID	Chr	Position	Genes	FAM primer sequence (5'>3')	HEX primer sequence (5'>3')	Reverse primer sequence (5'>3')
rs723240647	3	15079217	*GON4L*	CAACGCTGGATAAGTCTTCGGC	CAACGCTGGATAAGTCTTCGGG	TGCTTAGCAGCCCTCTGGGGAA
rs715250609	3	16131785	*TDRD10*	GTCAATTGTATTTCTATACAGAGGGGA	CAATTGTATTTCTATACAGAGGGGG	CTGTGTCTATGTCTTGCACAACTTTTATTA
