# Supplementary figures and images for: A frameshift mutation in GON4L is associated with proportionate dwarfism in Fleckvieh cattle
Source: Genet Sel Evol. 2016 Mar 31;48:25. doi: 10.1186/s12711-016-0207-z (PMC4818447; doi:10.1186/s12711-016-0207-z)

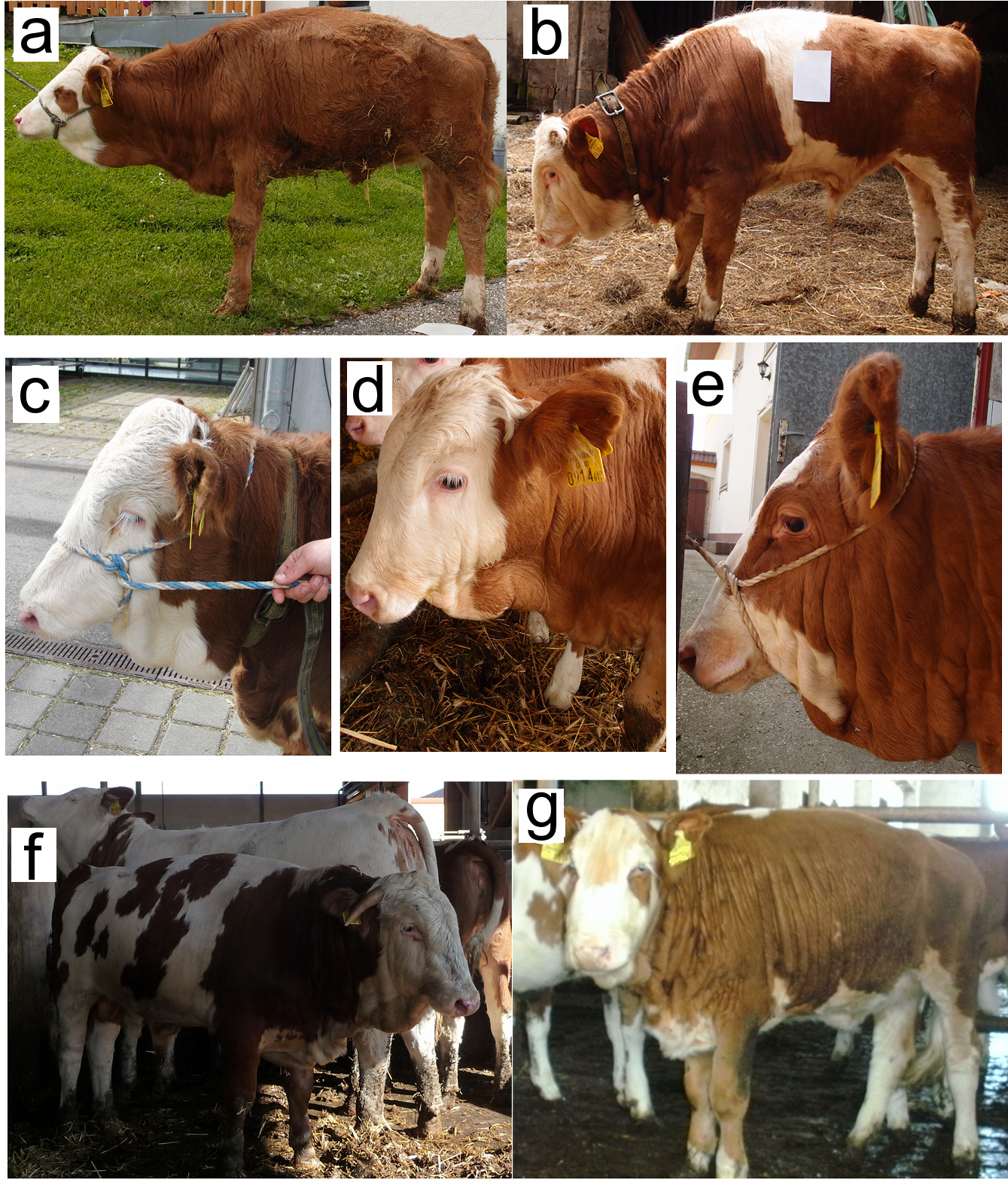

Supplement: Supplementary file 2 — 10.1186/s12711-016-0207-z Fleckvieh animals with dwarfism. Affected calves with a crooked back (a, b), elongated narrow heads and brachygnathia inferior (c-e). Wrinkled skin and areas with excessive skin (particularly in the neck area) became evident during rearing (c, e, g). A 19-month old animal with dwarfism and an 11-month old healthy animal (f). The head of the affected animal was disproportionately large compared to its body. [file 12711_2016_207_MOESM2_ESM.tif]

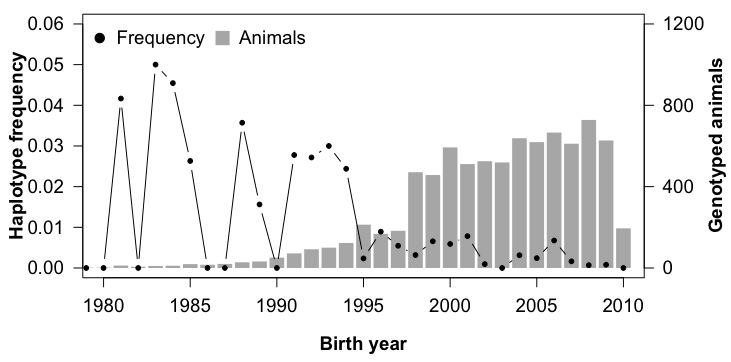

Supplement: Supplementary file 3 — 10.1186/s12711-016-0207-z Frequency of the DW-associated haplotype in 8332 Fleckvieh bulls. Grey bars and black dots represent the number of genotyped bulls and the haplotype frequency, respectively, per birth year. [file 12711_2016_207_MOESM3_ESM.png]

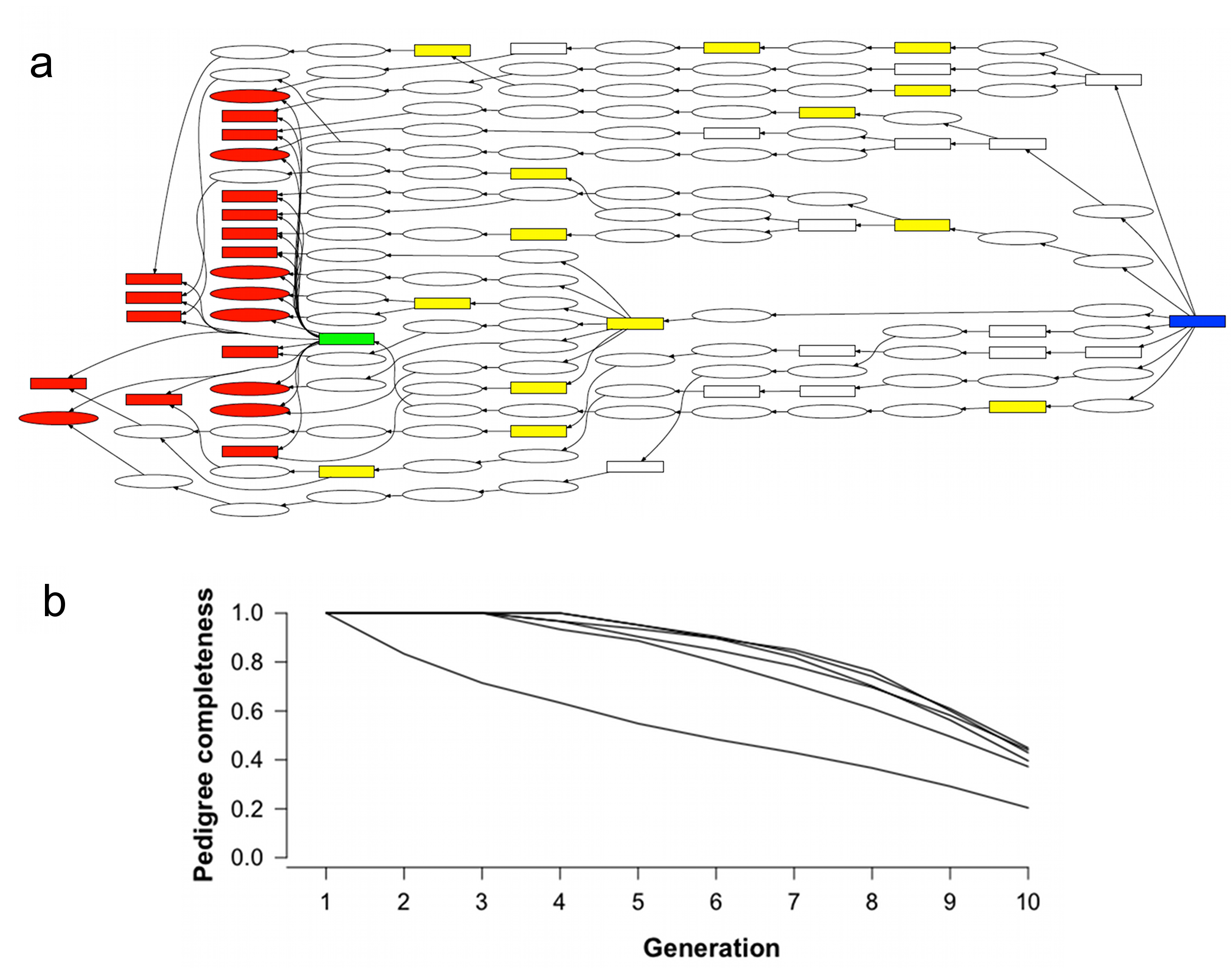

Supplement: Supplementary file 4 — 10.1186/s12711-016-0207-z Analysis of pedigree records of 27 animals with dwarfism. Description: Pedigree of 21 animals with dwarfism (a). The pedigree contains only obligate mutation carriers. Red and yellow colours indicate 21 affected animals and 14 genotyped haplotype carriers, respectively. Green and blue colours indicate the sire of 21 affected animals and DWhet, respectively. Rectangles and ovals represent male and female animals, respectively. Pedigree completeness of another six animals with dwarfism with no connection to DWhet on the maternal path (b). [file 12711_2016_207_MOESM4_ESM.tif]

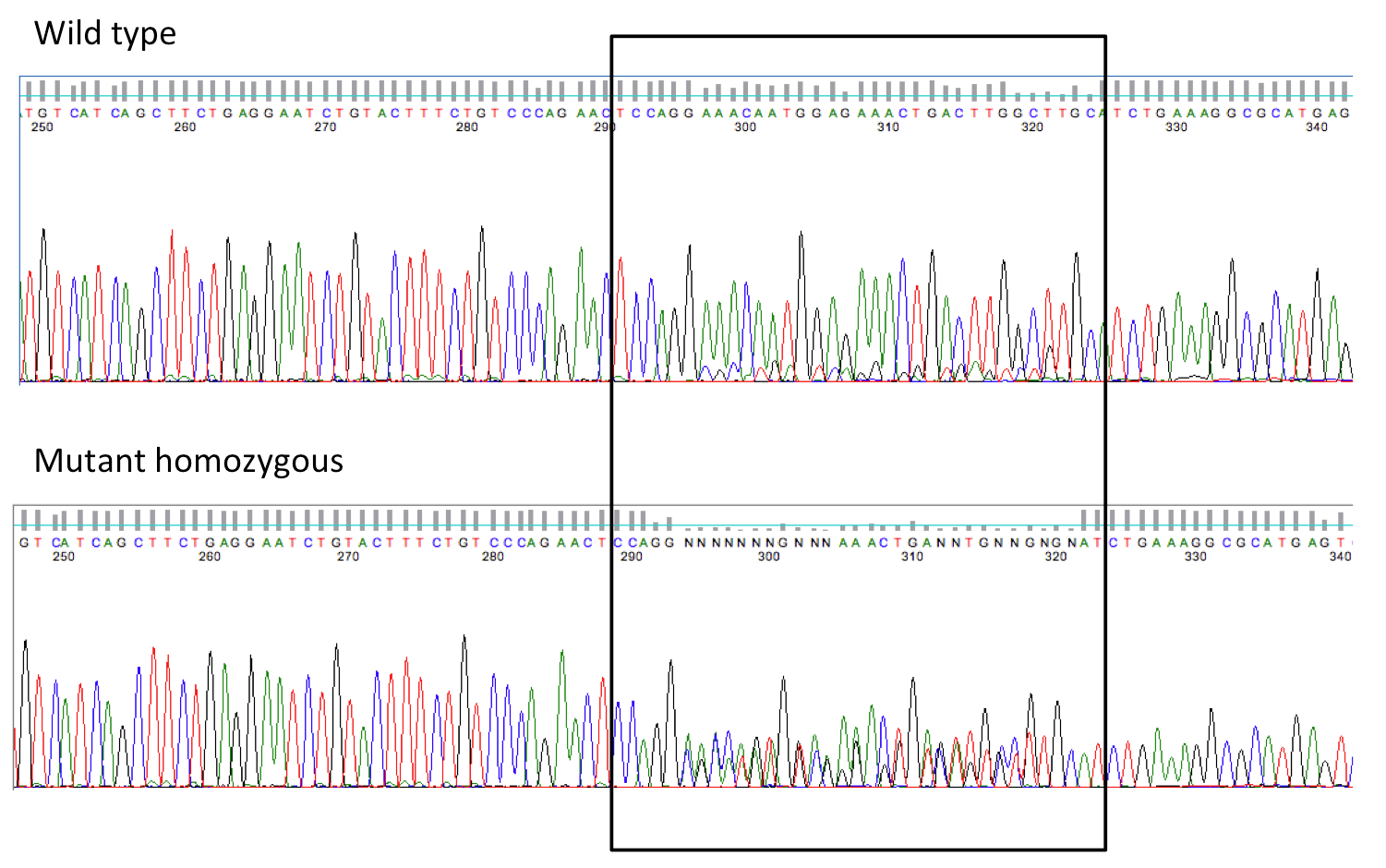

Supplement: Supplementary file 7 — 10.1186/s12711-016-0207-z Electrophoregram presenting the GON4L cDNA sequencing data with superimposed sequence at the 5’ terminal end of exon 21 (marked within a box). [file 12711_2016_207_MOESM7_ESM.png]
